# Randomized, Controlled Pilot Study of Low-Dose Human Chorionic Gonadotropin Administration Beginning From the Early Follicular Phase for Women With Polycystic Ovarian Syndrome Undergoing Ovarian Stimulation Using the Progesterone Protocol

**DOI:** 10.3389/fendo.2019.00875

**Published:** 2019-12-13

**Authors:** Xiuxian Zhu, Yonglun Fu

**Affiliations:** Department of Assisted Reproduction, Shanghai Ninth Peoples Hospital Affiliated to Shanghai Jiaotong University School of Medicine, Shanghai, China

**Keywords:** polycystic ovarian syndrome, progesterone protocol, human chorionic gonadotropin, frozen-thawed embryo transfer, preovulatory follicles, ovarian hyperstimulation syndrome

## Abstract

**Purpose:** To illustrate whether low-dose human chorionic gonadotropin (hCG) administration during the early follicular phase could reduce the number of large preovulatory follicles in women with polycystic ovarian syndrome (PCOS) undergoing ovarian stimulation using the progesterone protocol.

**Methods:** We performed a randomized, controlled pilot trial at a university-affiliated tertiary hospital. A total of 40 infertile women undergoing their first *in vitro* fertilization (IVF)/intracytoplasmic sperm injection (ICSI) treatment with the freeze-all strategy were included. Human menopausal gonadotropin (hMG) and progesterone soft capsule 100 mg/d were added simultaneously beginning from menstrual cycle day 3 for all participants. Low-dose hCG (200 IU) was injected every 3 days in the study group from the first day of ovarian stimulation until trigger. The primary outcome was the number of large preovulatory follicles. Secondary outcomes included the incidence of ovarian hyperstimulation syndrome (OHSS); the number of oocytes retrieved, mature oocytes, and good-quality embryos; and clinical results after frozen-thawed embryo transfer (FET) cycles.

**Results:** The study group had slightly more large preovulatory follicles than the control group (17.75 ± 10 vs. 13.2 ± 5.34; *P* > 0.05). None of the participants experienced severe OHSS. There were no statistically significant differences in the number of oocytes retrieved (15.9 ± 8.46 vs. 15.75 ± 6.96), mature oocytes (13.55 ± 6.56 vs. 13.4 ± 6.34), and good-quality embryos (5.5 ± 3.41 vs. 4.9 ± 2.99) between the two groups (*P* > 0.05). Clinical pregnancy rates (65.52 vs. 41.94%; *P* = 0.067) and live birth rates (48.28 vs. 35.48%; *P* = 0.315) per transfer following FET of the study group were higher than those of the control group, but without statistical significance.

**Conclusions:** Administration of low-dose hCG from the early follicular phase for PCOS patients undergoing ovarian stimulation with progesterone protocol may lead to slightly more early preovulatory follicles and marginally, but not significantly, higher clinical pregnancy rates. A continuous trial should be performed to explore the effects of supplementation with different doses of hCG from the start of ovarian stimulation in PCOS patients using the progesterone protocol.

**Clinical Trial Registration:** Chictr.org.cn, identifier: ChiCTR-IOR-15007165

## Introduction

Although the use of exogenous follicle-stimulating hormone (FSH) to promote multiple follicular development to yield multiple oocytes is well-established in assisted reproductive technology (ART), the addition of luteinizing hormone (LH) activity preparation remains controversial during ovarian stimulation. In prior decades of *in vitro* fertilization (IVF) treatments, it was considered that LH activity contained in human menopausal gonadotropin (hMG), which is mainly derived from human chorionic gonadotrophin (hCG), was detrimental to follicle growth and oocyte development during ovarian stimulation (1). However, studies have reported that the pregnancy and live birth rates were increased by using highly purified human menopausal gonadotrophins (HP-hMG) compared with recombinant human FSH (r-FSH), with improved high-quality embryos, different hormone dynamics, and different follicular patterns (1).

Excessive multiple follicle development is primarily responsible for ovarian hyperstimulation syndrome (OHSS) in women with polycystic ovarian syndrome (PCOS) during ovarian stimulation. The presence of many small-diameter antral follicles before trigger was associated with a higher rate of OHSS (2). Filicori et al. confirmed that low-dose hCG can be used to stimulate the growth of large follicles and accelerate the demise of small follicles (<10 mm diameter) when administered during the mid-follicular or late follicular phase of ovarian stimulation with the gonadotropin-releasing hormone agonist (GnRH-a) protocol or the GnRH antagonist (GnRH-ant) protocol (3–7), resulting in a lower incidence of OHSS (8). Additionally, it was demonstrated that the number of small preovulatory ovarian follicles was inversely correlated with the hCG dose administered (4).

Recently, we demonstrated that PCOS patients using the progesterone protocol could achieve competent oocytes/embryos and optimal pregnancy outcomes with frozen-thawed embryo transfer (FET). Based on the freeze-all strategy, progesterone agents could be provided as effective alternatives to GnRH-a and GnRH-ant for ovarian stimulation to modulate pituitary LH secretion (9–13), with the advantages of oral administration, user convenience, and reduced cost (10). Until now, no study had investigated hCG supplementation beginning in the early follicular phase with the progesterone protocol. In the current study, we attempted to determine whether low-dose hCG administration could reduce the number of large preovulatory follicles by restricting the follicular development of antral follicles and to elucidate the clinical outcomes of PCOS patients undergoing ovarian stimulation with the addition of hCG or without early follicular phase hCG administration.

## Materials and Methods

### Study Design and Participants

This randomized, controlled pilot study was performed at the Department of Assisted Reproduction of Ninth People's Hospital affiliated to Shanghai Jiao Tong University School of Medicine from July 2015 to December 2017. The trial was approved by the Institutional Review Board of the Ninth People's Hospital. All participating patients and their spouses signed informed consent relevant to infertility treatments with IVF/ICSI procedures.

The diagnosis of PCOS was made according to the 2003 Rotterdam criteria. Women with PCOS who were younger than 38 years and had a body mass index (BMI) <28 kg/m^2^ and planning to undergo treatment with IVF/intracytoplasmic sperm injection (ICSI) with the freeze-all strategy were eligible to participate. Women with a history of IVF/ICSI, severe endometriosis (grade 3 or higher), significant systemic disease, or other situations unsuitable for ovarian stimulation were excluded from the study.

### Study Protocol

Progesterone 100-mg soft capsules (Utrogestan; Laboratories Besins International, Montrouge, France) were orally delivered daily from menstrual cycle (MC) day 3, and 150 IU of hMG was administered concomitantly to all participants. Additionally, low-dose hCG (200 IU) was injected every 3 days in the study group from the first day of ovarian stimulation until trigger. During our trial, the study group was administered the HCG+HMG+Utrogestan protocol and the control group was administered the HMG + Utrogestan protocol.

Oocyte maturation was induced with 0.1 mg GnRH-a (Decapeptyl; Ferring Pharmaceuticals, Heidelberg, Germany) when there were more than three dominant follicles with a diameter of 18 mm. Transvaginal ultrasound-guided follicle aspiration was performed 36–38 h after trigger. Fertilization, embryo assessment, and endometrial preparation for FET were performed using routine procedures (12).

### Randomization and Sample Size Estimate

Subjects were randomly assigned in a 1:1 ratio to the study group or the control group via a sealed envelope with random numbers generated by a computer. Participants were not blinded to the group assignment. The physicians and embryologists involved in the oocyte retrieval and embryo transfer were blinded to the group assignments of the participants in the trial.

The pilot study was conducted to explore the benefits of different treatment methods using an adaptive design. With the adaptive design, a single trial could be separated into two stages. The study stopped after stage 1 or the study continued to stage 2, during which the planned design could be modified and the sample size for stage 2 could be calculated based on the results of stage 1 (14). Lachin et al. proposed that the least number of patients required for such studies using an adaptive design was 15 (15). Because of the drop-out rate, we included 20 patients in each group in our pilot study.

### Outcome Measures

The primary outcome measure was the number of large preovulatory follicles (follicles with a diameter >14 mm on the trigger day). Secondary outcome measures included the following: the occurrence of severe OHSS and premature LH surge; dynamic characteristics of steroid hormones; duration and dosage of hMG; the numbers of oocytes retrieved, mature oocytes, and good-quality embryos; the rate of OHSS; clinical pregnancy rate; implantation rate; miscarriage rate; and live birth rate.

Clinical pregnancy was defined as the presence of a gestational sac with or without heart activity as assessed at least 35 days after FET. Live birth was defined as the delivery of an infant after 28 weeks of gestation.

### Hormone Analysis

The chemiluminescent method (Abbott Biologicals B.V., Olst, the Netherlands) was adopted to measure hormone levels. The intra-assay and inter-assay coefficients of variation, respectively, were as follows: 2.6% and 5.8% for FSH; 5.9% and 8.1% for LH; 6.3% and 6.4% for estradiol (E_2_); and 7.9% and 10% for progesterone. The lower limits of sensitivity were 0.06 IU/L for FSH, 0.09 IU/L for LH, 10 pg/mL for E_2_, and 0.1 ng/mL for progesterone. Furthermore, 5,000 pg/mL was the upper limit for E_2_ measurements; in other words, it was recorded as 5000 pg/mL if the E_2_ levels were higher than the upper limit.

### Statistical Analysis

Efficacy analyses were based on the intent-to-treat population, which was defined as all women randomized to receive the progesterone protocol with or without low-dose hCG administration. Continuous variables are presented as mean ± standard deviation (SD), and categorical variables are shown as number and percentage. Student's *t*-test was used for normally or near-normally distributed data, and the Mann-Whitney U test was used for non-normally distributed data for the comparison of continuous variables. Categorical variables were compared with the chi-square test and Fischer's exact test. Two-sided *P* < 0.05 was defined as statistically significant. Statistical Package for the Social Sciences for Windows version 24.0 was used for analysis (SPSS, Chicago, IL, USA).

## Results

### Patient Characteristics

**Figure 1:**
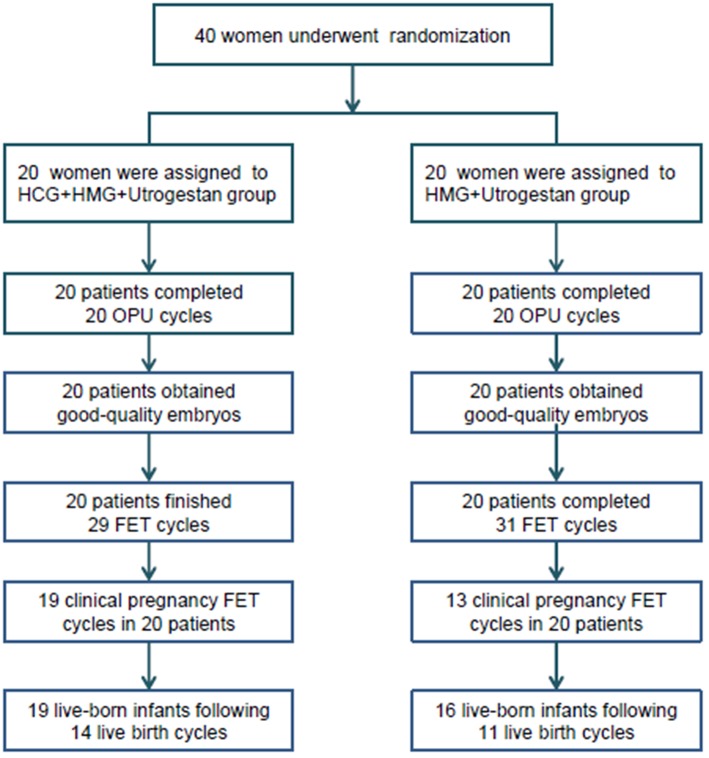
Flowchart of the study. OPU, oocytes pick-up; FET, frozen-thawed embryo transfer; HCG, human chorionic gonadotropin; HMG, human menopausal gonadotropin.

A flowchart of the study is presented in Figure 1. A total of 40 women were included, among which the first participant was recruited in November 2015, and the last participant was recruited in November 2017. Good-quality embryos were obtained from all participants who underwent oocyte retrieval, and a total of 60 FET cycles were completed during the follow-up period. There was no statistical difference in the general characteristics of the patients (Table 1).

**Table 1:** The basic characteristics of women in the trial.

**Characteristic**	**Study group (HCG+HMG+** **Utrogestan protocol)**	**Control group (HMG+** **Utrogestan protocol)**	***P*-value**
Age (years)	29.35 ± 3.76	31.05 ± 3.8	0.106
BMI (kg/m^2^)	22.9 ± 2.24	23 ± 2.53	0.672
Duration of infertility(years)	3.35 ± 1.35	4.3 ± 3.05	0.739
Antral follicle counts(*n*)	28.65 ± 6.65	28.6 ± 4.88	0.978
Indication, *n* (%)			0.801
PCOS only	4	3	
PCOS + tubal factor	10	13	
PCOS + male factor	2	2	
PCOS + combined factors	4	2	
Basal FSH (IU/L)	4.86 ± 1.34	5.09 ± 0.87	0.219
Basal LH (IU/L)	8.37 ± 6.79	7.98 ± 4.56	0.802
Basal E2 (pg/mL)	37.95 ± 14.46	38.82 ± 12.55	0.707
Basal P (ng/mL)	0.29 ± 0.14	0.29 ± 0.17	0.851

*HCG, human chorionic gonadotropin; HMG, human menopausal gonadotropin; Utrogestan, brand name of a type of natural micronized progesterone; BMI, body mass index; PCOS, polycystic ovarian syndrome; FSH, follicle-stimulating hormone; LH, luteinizing hormone; E2, estradiol; P, progesterone*.

### Outcomes of Ovarian Stimulation

Table 2 shows the outcomes of ovarian stimulation for both groups. The total hMG dose (1642.5 ± 728.74 vs. 1856.25 ± 1192.41 IU) was slightly less and the mean hMG duration (9.3 ± 2.94 vs. 10.25 ± 3.75) was shorter for the study group than for the control group, with no significant difference (*P* > 0.05). Additionally, the number of large preovulatory follicles (17.75 ± 10 vs. 13.2 ± 5.34, *P* = 0.167) and the number of follicles with a diameter >10 mm in the study group were slightly greater than those in the control group (20.15 ± 9.21 vs. 16.1 ± 5.88, *P* = 0.184), with no statistical difference. In the study group, 15.9 ± 8.46 oocytes were retrieved; in the control group, 15.75 ± 6.96 oocytes were retrieved (*P* = 0.828). There were no statistically significant differences in the numbers of mature oocytes (13.55 ± 6.56 vs. 13.4 ± 6.34) and good-quality embryos (5.5 ± 3.41 vs. 4.9 ± 2.99) for the two groups (*P* > 0.05). Similarly, the two groups were comparable regarding the numbers of fertilized oocytes, cleaved embryos, and day 3 high-quality embryos. Severe OHSS was not observed in either group during the study.

**Table 2:** The cycle characteristics and embryo results of ovarian stimulation in two regimens.

**Characteristic**	**Study group (HCG+HMG** **+Utrogestan protocol)**	**Control group** ** (HMG+Utrogestan protocol)**	***P*-value**
hMG duration (days)	9.3 ± 2.94	10.25 ± 3.75	0.262
hMG dose (IU)	1642.5 ± 728.74	1856.25 ± 1192.41	0.924
FSH on trigger day (IU/L)	11.07 ± 2.65	13.09 ± 4.2	0.158
LH on trigger day (IU/L)	4.41 ± 3.49	3.91 ± 2.02	0.965
E2 value trigger day(pg/mL)	4441.15 ± 937.32	4022.56 ± 1249.23	0.460
P value on trigger day (ng/mL)	5.23 ± 2.26	5.09 ± 2.9	0.393
No. of >10 mm follicles on trigger day	20.15 ± 9.21	16.1 ± 5.88	0.184
No. of >14 mm follicles on trigger day(large)	17.75 ± 10	13.2 ± 5.34	0.167
No. of oocytes retrieved	15.9 ± 8.46	15.75 ± 6.96	0.828
No. of MII oocytes	13.55 ± 6.56	13.4 ± 6.34	0.818
No. of oocytes fertilized	12.05 ± 6.76	11.45 ± 5.05	0.968
No. of cleaved embryos	11 ± 5.96	9.95 ± 4.3	0.765
No. of Day 3 top-quality embryos	3.75 ± 2.27	3.95 ± 2.33	0.934
No. of good-quality embryos	5.5 ± 3.41	4.9 ± 2.99	0.723

*HCG, human chorionic gonadotropin; HMG, human menopausal gonadotropin; Utrogestan, brand name of a type of natural micronized progesterone; BMI, body mass index; PCOS, polycystic ovarian syndrome; FSH, follicle-stimulating hormone; LH, luteinizing hormone; E2, estradiol; P, progesterone*.

### Pregnancy Outcomes Following Frozen-Thawed Embryo Transfer

As illustrated in Table 3, participants in the two groups transferred similar numbers of embryos. The clinical pregnancy rate per transfer (65.52 vs. 41.94%; *P* = 0.067) and implantation rate (47.17 vs. 35.09%; *P* = 0.198) were higher in the study group than in the control group, without significant differences. The rates of biochemical pregnancy, early miscarriage, and late miscarriage were also comparable between the two groups. The birth weights of singleton newborns and twin newborns in the two groups were not statistically different.

**Table 3:** Pregnancy and live birth outcomes of frozen-thawed embryos originating from the two regimens.

**Outcomes**	**Study group (HCG+HMG+** **Utrogestan protocol)**	**Control group (HMG+** **Utrogestan protocol)**	***P-*value**
Patients (*n*)	20	20	
FET cycles (*n*)	29	31	
No. of thawed embryos (*n*)	53	57	
No. of transferred embryos per FET cycle (*n*)			1.000
With one embryo	5	5	
With two embryos	24	26	
No. of remaining frozen embryos	57	41	
No. of patients with surplus embryo frozen	11	12	
Biochemical pregnancy rate per transfer % (*n*)	68.97% (20/29)	51.61% (16/31)	0.17
Clinical pregnancy rate per transfer% (*n*)	65.52% (19/29)	41.94% (13/31)	0.067
Clinical pregnancy rate per patient % (*n*)	95% (19/20)	65% (13/20)	0.044
Implantation rate% (*n*)	47.17% (25/53)	35.09% (20/57)	0.198
Early miscarriage rate % (*n*)	5.26% (1/19)	7.69% (1/13)	1.000
Late miscarriage rate % (*n*)	10.53% (2/19)	7.69% (1/13)	1.000
Ectopic pregnancy % (*n*)	10.53% (2/19)	0% (0/13)	0.502
Live birth per cycle % (*n*)	48.28% (14/29)	35.48% (11/31)	0.315
Live birth per patient % (*n*)	70% (14/20)	55% (11/20)	0.327
Newborn			
Single birth (*n*)	9	6	
Single birthweight (g)	3183.33 ± 343.03	3251.67 ± 334.39	0.731
Twin birth (*n*)	10	10	
Twin birthweight (g)	2691.67 ± 224.54	2575 ± 38.93	0.382

*HCG, human chorionic gonadotropin; HMG, human menopausal gonadotropin; Utrogestan, brand name of a type of natural micronized progesterone; FET, frozen-thawed embryo transfer*.

### Dynamic Hormone Levels During Ovarian Stimulation

**Figure 2:**
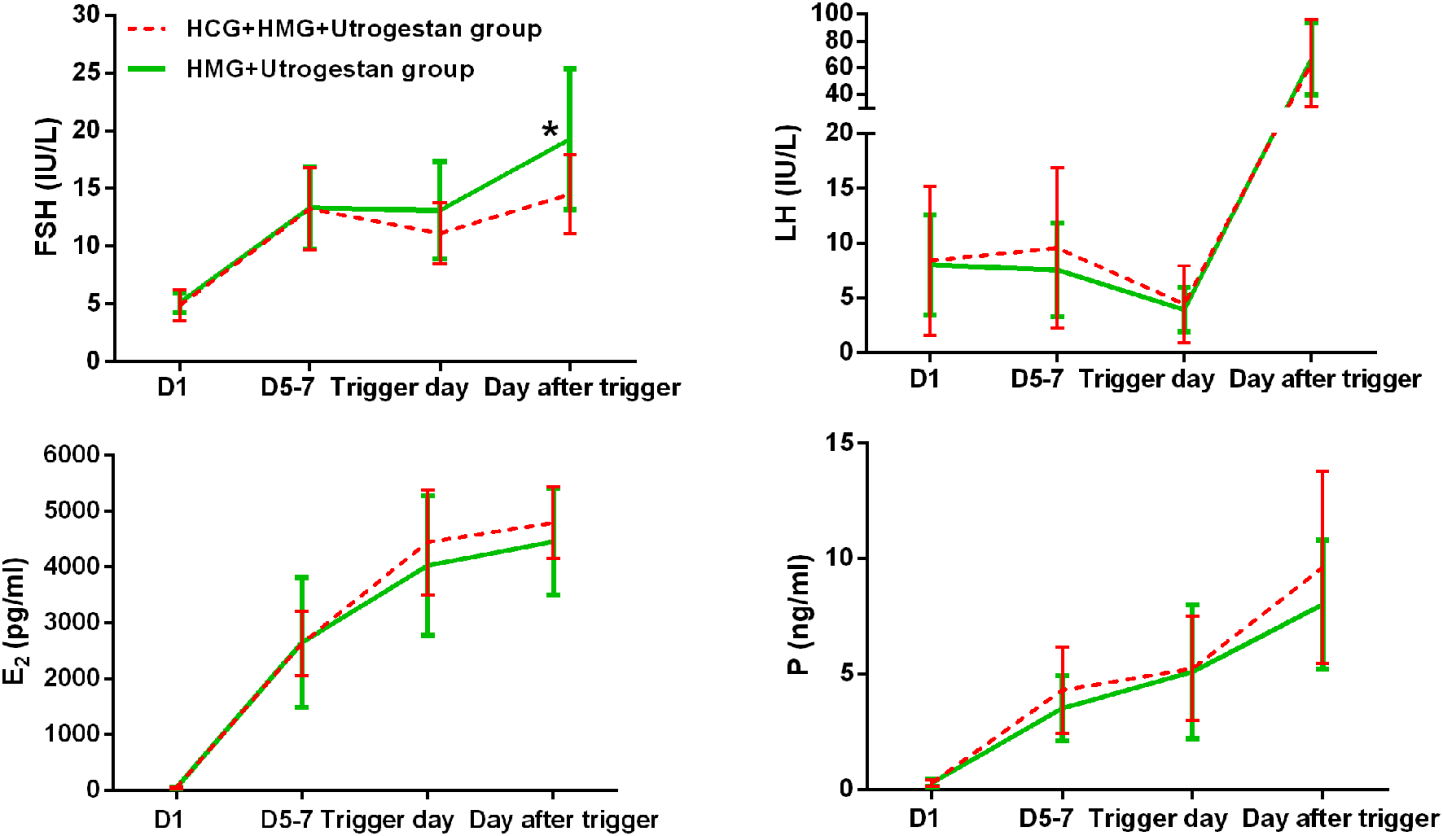
Serum hormone profiles during ovarian stimulation in the two groups. The green lines represent the control group (HMG+Utrogestan group). The red lines represent the study group (HCG+HMG+Utrogestan group). *Time point when *P* < 0.05. The starting day of ovarian stimulation is denoted as day 1 (D1). FSH, follicle-stimulating hormone; LH, luteinizing hormone; E_2_, estradiol; P, progesterone.

Table 2 shows the serum hormone levels on the trigger day for all participants, with no statistical difference. Figure 2 shows the changes in the trends of serum FSH, LH, E_2_, and progesterone values during the follicular phase in the two groups. The starting day was denoted as day 1 (D1). The FSH levels on D5 to D7 and on the trigger day were comparable in the two groups; however, they decreased below normal levels in the control group on the day after the trigger (*P* < 0.05). Average LH levels stayed comparable throughout the process of ovarian stimulation in the two groups. A premature LH surge was not detected in any participants. The average E_2_ of the study group on D5 to D7 was comparable with that of the control group; then, it increased above normal levels in the control group on the trigger day and on the day after the trigger (*P* > 0.05). Serum progesterone levels of the study group were similar to those of the control group throughout the ovarian stimulation process (*P* > 0.05).

## Discussion

In the present study, we tested whether early follicular phase low-dose hCG administration reduced the number of large preovulatory follicles. Our preliminary results showed that the large preovulatory follicles were not reduced; instead, they slightly increased with the introduction of low-dose hCG from the beginning of ovarian stimulation using the progesterone protocol when compared to the standard progesterone protocol without exogenous hCG administration.

Low-dose hCG administration from the start of stimulation is not a new procedure; it has been reported mainly for non-PCOS patients using the GnRH-a or GnRH-ant protocol (14, 16–18). A prospective, randomized, pilot study by Drakakis et al. compared low-dose hCG (200 IU) with rLH (200 IU) added to rFSH from the first 5 days of ovarian stimulation with the GnRH-ant protocol (16). Patients in the low-dose hCG group achieved more oocytes and higher implantation rates than the rLH group; however, the overall implantation rate was low for all participants, especially those in the LH group (16). It is known that a more estrogenic environment in the follicular fluid is beneficial for embryo quality; however, increasing doses of hCG supplementation may result in a shift toward a more androgenic intrafollicular environment (18), indicating that doses of hCG over a possible optimal dose may cause potentially harmful effects. To define an adequate dose of hCG that could be added throughout stimulation, Thuesen et al. conducted a prospective, randomized dose-response (0–150 IU/d) study that concluded that hCG supplementation up to a daily dose of 100 IU was recommended for patients treated with the GnRH-a protocol (14, 18). After combining those observed biochemical changes induced by hCG and the serum half-life of 2.32 days for hCG (19), we adopted 200 IU hCG every 3 days to maintain a consistent serum hCG level throughout ovarian stimulation.

It has been shown that low-dose hCG administration during the late follicular phase in the GnRH-a and GnRH-ant protocols could cause small (<10 mm diameter) preovulatory follicles atresia (3–7). Animal studies may provide an explanation of such an interesting phenomenon. For example, it was shown that the normal progression of folliculogenesis could be inhibited by excess androgen administration in monkeys (20). In the rat, the addition of LH prevented the ovulatory response induced by FSH in a dose-related manner (21). The administration of anti-androgens or anti-testosterone antiserum reversed the effects of ovarian weight increments and the rate of multi-follicular progress resulting from the addition of hCG (22, 23). According to the concept of androgen-mediated atresia of small follicles, we expected that the addition of hCG from the first day of stimulation in our trial would reduce the number of large preovulatory follicles by restricting the follicular development of antral follicles. However, there were more large preovulatory follicles and follicles with diameters >10 mm in the study group than in the control group, despite there being no statistically significant differences. This was in accordance with an RCT involving patients with normal ovulation using a modified GnRH-ant protocol that reported that the proportion of antral follicles reaching ≥14 mm was higher in the study group (24). Several animals studies have indicated that the sensitivity of granulosa cells to FSH could be increased with the addition of androgen, which may improve follicular responsiveness (25); additionally, the number of small antral follicles could be increased by androgens and the proliferation of granulosa and theca cells (26, 27). Clear knowledge of the threshold at which androgen begins to restrict the number of small follicles is lacking. We proposed that there may be an optimal dose of hCG that could be used with early follicular phase hCG administration to achieve atresia of the antral follicles and reduce the number of large preovulatory follicles in PCOS patients.

Additionally, we compared clinical outcomes of PCOS patients using the progesterone protocol with or without the addition of low-dose hCG from the first day of ovarian stimulation. The numbers of retrieved oocytes, mature oocytes, and good-quality embryos were similar between the two groups, but the clinical pregnancy rate and implantation rate of the study group were higher than those of the control group (no statistical difference). These results were in accordance with those of a retrospective observational study in which higher implantation and pregnancy rates were obtained despite fewer oocytes and fewer embryos in the study group than in the rFSH alone group (17). The direct effect of hCG on endometrium receptivity was once speculated to result in better pregnancy results when embryo transfer was scheduled after oocyte retrieval. A recent study showed that implantation and pregnancy rates were significantly increased by an intrauterine injection of hCG 500 IU before transfer (28). However, good-quality embryos from all participants were frozen during the current study and saved for later transfer, which excluded the possible impact of hCG on the endometrium. The intrafollicular concentrations of estrogen and progesterone were positively associated with embryo quality. The ratio of E_2_ to progesterone was significantly and inversely related to good outcomes, indicating that the most competent oocytes were generated from follicles involved in the timely transition to progesterone production (18). Even though the addition of hCG seemed to increase E_2_ and progesterone levels, a statistical difference was not achieved, possibly due to the limited sample size and upper limit of 5,000 pg/mL for E_2_ measurements. We could not find evidence for the potential positive impact of hCG on the development of competent embryos in our study. The existence of a possible trend toward higher clinical pregnancy rates for patients who received low-dose hCG during the early follicular phase may be attributable to the limited population. Therefore, further clinical trials with large sample sizes and more homogenous participants are required.

This trial had several limitations. First, the limited sample size was a drawback of the study. Because the addition of hCG had not been previously used from the first day of stimulation with the progesterone protocol, we tested the effects of this treatment in a small group as a pilot trial before a larger trial was conducted. Second, the gonadotropin used in our trial was hMG instead of rFSH because of the cost, and all participants were administered the same type of gonadotropin, which might have reduced the potential bias of hCG components contained in hMG. Third, at the end of the trial, there were 98 remaining frozen embryos waiting for transfer, so the cumulative pregnancy rate per patient was still conservative. The slightly better clinical pregnancy outcomes of the study group were probably due to the small sample size and chance. Finally, the open-label design, which allowed patients to know their treatment assignment, may have been a limitation; we compensated for this potential bias by blinding the physicians and embryologists involved in the trial.

Our preliminary assumption was that low-dose hCG administration beginning from the early follicular phase could reduce the number of large preovulatory follicles during ovarian stimulation. However, our data showed there were more large preovulatory follicles in the study group than in the control group. Due to the unresolved threshold for androgen-mediated atresia of small follicles, the adequate dose of hCG remains to be elucidated for clinical use. Therefore, we decided to terminate this trial after stage 1 and investigate the effects of supplementation with different doses of hCG in our subsequent studies.

## Conclusion

Our results showed that the use of low-dose hCG from the beginning of ovarian stimulation in women with PCOS using the progesterone protocol may lead to slightly more early preovulatory follicles. Furthermore, we presented marginally, but not significantly, higher clinical pregnancy rates during this pilot study. The results of our pilot study should be considered preliminary instead of as final conclusions for clinical practice. A continuous trial should be performed to explore the effects of supplementation with different doses of hCG from the start of ovarian stimulation in PCOS patients using the progesterone protocol.

## Data Availability Statement

The datasets generated for this study are available on request to the corresponding author.

## Ethics Statement

This study received Human Ethics Committee approval from the Institutional Review Board (IRB) of the Shanghai Ninth People's Hospital on 6 July 2015 (number: 2015-55). Chinese clinical trial registration number: ChiCTR-IOR-15007165 (www.chictr.org.cn/showproj.aspx?proj=12056). All participants have signed informed consent for this research.

## Author Contributions

YF was responsible for the conception, design, and completion of the entire study. XZ collected the data, contributed to the analysis of data, and wrote the manuscript.

### Conflict of Interest

The authors declare that the research was conducted in the absence of any commercial or financial relationships that could be construed as a potential conflict of interest.
